# Effect of 3D Animation Combined with Teach-Back Health Education on Pelvic Floor Muscle Training in LARS Patients: A Randomized Controlled Trial

**DOI:** 10.1155/2023/6847933

**Published:** 2023-02-24

**Authors:** Jingjing Ye, Xiaoyan Xu, Shengnian Lu, Xiaojun Xu, Hanmei Liu, Mingxian Luo, Jiamei Zhou, Lianhong Wang, Yongmei Zhang

**Affiliations:** ^1^Department of Cardiovascular Surgery, Affiliated Hospital of Zunyi Medical University, Zunyi, Guizhou, China; ^2^Affiliated Hospital of Zunyi Medical University, Zunyi, Guizhou, China

## Abstract

**Aim:**

The present study aimed to evaluate the effect of 3D animation combined with teach-back health education on the recovery of low anterior resection syndrome (LARS) patients.

**Background:**

LARS is the most common problem after anus-preserving surgery in rectal cancer. Pelvic floor muscle training can promote the recovery of recto-anal function.

**Methods:**

Ninety-nine patients with LARS were randomly divided into control group, experiment group I, and experiment group II. The control group was guided by one-to-one verbal pelvic floor muscle training. The experiment group I was given self-made 3D animation along with one-to-one pelvic floor muscle guidance. The 3D animation and teach-back methods were used for training and guidance in the experiment group II. The outcome measures were scores of low anterior resection syndrome scale items, as well as the completion of training content.

**Results:**

The degree of completion of training content in the experiment group II was higher than that of the control group and experiment group I. The total score of LARSS in the experiment group II was significantly lower than in the control group and experiment group I. In particular, scores of loose stool incontinence, defecation frequency level, tenesmus, and defecation urgency in experiment group II were better than those in the control group.

**Conclusion:**

3D animation combined with teach-back health education improved the mastery of pelvic floor muscle training theory and practice in LARS patients, and effectively reduced the symptoms. *Implications for Nursing Management*. This intervention promoted the recovery of pelvic floor muscle function in LARS patients and can be regarded as an effective measure to improve quality of life and provide better clinical care for patients.

## 1. Background

According to pertinent statistical reports, the incidence and mortality of colorectal cancer rank third and second in the world, respectively, and it has become the most prevalent malignant tumor of the digestive system in China, among which rectal cancer accounts for about 60%–75% [1, 2]. At present, the treatment methods for rectal cancer mainly include combined abdominoperineal resection (Miles) and transabdominal resection and anastomosis (Dixon), also known as anal preserving surgery, accounting for 70% of the surgical methods [3]. Following anus-preserving surgery, 90% of patients show a series of defecation-related bowel function changes such as increased defecation frequency, difficulty in excretion, and incontinence, a phenomenon referred to as low anterior resection syndrome (LARS) [4, 5].

At present, the treatment for LARS consists of drug therapy (tryptamine agonists and antagonists), anal lavage and anterograde enema, sacral nerve stimulation and percutaneous tibial nerve stimulation, pelvic floor rehabilitation training (pelvic floor muscle exercise, rectal balloon training, and biofeedback training), and other methods [6, 7]. Pelvic floor muscle training refers to the voluntary contraction of pelvic floor muscles, dominated by the levator ani muscle, in a conscious and rhythmic way to strengthen defecation control ability, and is simple and easy to learn, while not being restricted by external objective conditions [8]. At present, pelvic floor muscle training is widely used in the treatment of stress urinary incontinence, postpartum urinary retention, postpartum pelvic floor function, and sexual dysfunction [9, 10], and the effects are significant.

The vivid image characteristics of 3D animation creates a rich color saturation viewing experience, increasing and improving learners' perceptual understanding [11]. Teach-back is a communication method that allows information recipients to repeat or demonstrate the information provided to them using their own language and actions to confirm their understanding. The technique comprises four steps: explanation, evaluation, clarification, and understanding [12]. Studies have shown that this feedback method has significant effects in reducing the readmission rate of patients, improving diet compliance, and reducing surgical complications and medication error rates [13]. At present, the study of feedback methods in Chinese health education focuses only on chronic diseases, and there are no reports of its impact on intestinal function and quality of life in patients with rectal cancer LARS.

Therefore, this study proposes a training program for LARS patients to quickly recover pelvic floor muscle function, which can improve their quality of life, and also provide a reference for medical staff to provide them with appropriate care.

## 2. Methods

### 2.1. Program Development

This study used a randomized controlled trial to evaluate the effectiveness of the developed program. In the control group, the responsible nurse selected a one-to-one verbal health education method to provide patients with pelvic floor muscle knowledge and guidance on training the muscles. Group I conducted one-to-one education with the vivid, three-dimensional 3D animation, and training measures the same as those in the control group. In group II, the patients were trained with 3D animation and teach-back (pelvic floor muscle training method guidance, assessment of mastery, repeat of guidance deficits, confirmation of mastery, and continuous exercise).

### 2.2. Design and Study Protocol

Patients enrolled in the study were randomly divided into three groups using SPSS Statistics for Windows, version 18.0 random number generator program. The results of randomization were 33 cases in the control group, 33 cases in the experiment group I, and 33 cases in the experiment group II. To ensure validity, this study was designed as a single-blind trial.

### 2.3. Outcome Measures

All the patients in the three groups started training on the 7th day after surgery which lasted till 3 months after. Training took place daily at 8 AM, 2 PM, and 6 PM. The baseline data recorded comprised the low anterior resection syndrome score (LARSS) and the training content completion evaluation.

### 2.4. Participants and Setting

Participants were LARS patients from the gastrointestinal surgery department of Guizhou Provincial Class A Hospital. The age range included in this study was 45 to 75 years. They were diagnosed with rectal cancer and first treated with anus-preserving surgery. The LARSS scale at baseline was scored at ≥21, with the rating level indicating above mild severity of LARS [14]. Participants chosen had constant access to their mobile phone during hospitalization and discharge and had a long-term primary caregiver (care time of no less than 4 h/d; if there were many caregivers, the longest care time was considered; caregivers included family members who were ≥18 years of age and were fluent in the language) [15].

### 2.5. Procedures

#### 2.5.1. The Control Group

The responsible nurse engaged the patient in one-to-one verbal health education to provide them with relevant, pelvic floor muscle knowledge and guidance on training the muscles before surgery, after surgery, on the day of discharge, and when returning to the hospital for chemotherapy [16]. The muscles of pelvic floor muscle training mainly include the levator anal muscle, the internal and external anal sphincter, the transverse and transverse anal canal muscle, and the coccygeal muscle.

#### 2.5.2. Experiment Group I

3D animation was used to enhance their knowledge of pelvic floor muscles and guide them in performing pelvic floor muscle training. The content and duration of the training were the same as in the control group.

A 3D motion capture camera was used to capture and save videos of the standardized patients. In the first part of the animation, Autodesk-Maya modeling software was used to recreate the pelvic floor muscle and bone model on the computer. In the second part, the muscle and bone model was superimposed on the corresponding anatomical position in the human body captured on video and presented with perspective three-dimensional effects. Finally, a complete 3D animation was made using the post-effect editing function of Adobe After Effects.

#### 2.5.3. Experiment Group II

The patients were trained using the 3D animation combined with teach-back. The training content and duration were the same as the control group. The intervention involved the following steps:

(1) The teach-back health education team was established. The head nurse of the department coordinated and managed the team, 2 responsible nurses were mentors of the education team, 1 subject researcher, and 1 gastrointestinal surgeon and 1 rehabilitation training instructor in the department served as health consultants.
(2) The 3D animation was played for the patients and their families to enhance the guidance and explanation.
(3) On a postoperative day 7: the teach-back procedures were used to continue the pelvic floor muscle training
  - Guidance on pelvic floor muscle training methods: the researchers watched the 3D animation again with the patients and reviewed the information and functional training methods shared before surgery.
  - Assessment of mastery: The patient was asked to repeat the procedure for pelvic floor muscle training in their own words and demonstrate it. The method explanation and the completion degree of the exercises by the patient was evaluated by the trainer, who maintained detailed records.
  - Repeated guidance for deficiencies: further guidance for deficiencies of the patient was given according to the records.
  - Mastery and continued use of the exercise are confirmed: mastery of the exercises was evaluated, and video playback and one-on-one education applied until the patient fully grasped the entire information and procedure.
(4) After discharge: every Monday, Wednesday, and Friday, mobile phone communication was used to remind patients to train, to understand their training situation, and give guidance.
(5) Returning to hospital for chemotherapy: patient's understanding and accuracy of practice was recorded and guidance was provided if necessary.

### 2.6. Ethical Considerations

This study was approved by the Medical Ethics Committee of the Affiliated Hospital of Guizhou Zunyi Medical University (review approval document no.KLLY-2020-143). During the intervention, the researcher strictly followed the bioethical principles of benefit and no harm, informed consent, confidentiality, ethics, and fairness.

### 2.7. Statistical Analysis

SPSS Statistics for Windows, version 18.0 was used to sort and analyze the data, and the level of significance was set at 5%. Unpaired *F* tests and the *χ*^2^ test were used to compare the participants' baseline data. The measurement data were presented by $\bar{x}$ *±* *s*, and the three groups of data, with normal distribution and homogeneity of variance, were tested by repeated measurement ANOVA. Before the analysis of variance, the sphericity test was performed for all of them, and if the results were not satisfactory, multivariate tests were conducted. After repeated measures of ANOVA, if there was an overall interaction, comparisons between groups and within groups were carried out. One-way ANOVA test was used for comparison, and the least significant difference (LSD) method was used for post-hoc multiple comparisons when significant differences were found. The Kruskal–Wallis H rank sum test was used in non-normal measurement data, and in the nonparametric test was used for rank data.

## 3. Results

### 3.1. Patients Baseline Data

A total of 99 patients with LARS were enrolled in this study, assigned as 33 patients each were to the control group, experimental group I, and experimental group II. In the course of the study, one case in the control group was discharged automatically in advance and one case could not be contacted after discharge. One case in experimental group I had poor compliance, one case in experimental group II had poor compliance, as well and one other case was transferred to another department for treatment. Finally, a total of 94 patients completed the program and data collection phase of the study. There were no significant differences in baseline data such as gender, age, height, weight, and BMI among the three groups (*P* > 0.05), indicating comparability. Specific comparison results are shown in Table 1.

### 3.2. Pelvic Floor Muscle Training Content Completion

When comparing the degree of completion of the training content, experiment group I and experiment group II were better than the control group before the surgery, and 7 days, 14 days, 1 month, and 3 months post-operation (*P* < 0.05). The details are shown in Table 2.

### 3.3. LARSS Total Score

The total score of LARSS for the three groups was analyzed and that of the experiment group II was significantly lower than that of the control group one month after the operation (*P* < 0.05). The total score of LARSS in the experiment II group was lower than that in the experiment I group and the control group at 3 months after the operation. There were statistically significant differences in the total score of LARSS between the control group and the experiment II group before and after intervention (*P* < 0.05). Specific comparison results are shown in Table 3.

### 3.4. Comparison of Scoring on Individual LARSS Items

The time effect of LARSS items was statistically significant (*P* < 0.05). The treatment effects of loose stool incontinence, frequency of defecation, and urgency of defecation were also statistically significant (*P* < 0.05). The interaction effects of loose stool incontinence, frequency of defecation, tenesmus-related weight loss, and urgency of defecation scores were statistically significant (*P* < 0.05). One-way analysis of variance was used to compare the three groups at different time points. The results showed that the defecation urgency score of group II was better than that of the control group on the 14th day after the operation; defecation frequency, 1 month after operation; loose stool incontinence, 3 months after operation; defecation frequency level, tenesmus, defecation urgency score in the experiment group II were consistently better than those in the experiment group I and control group. The scores of loose stool incontinence in experiment group II were better than those in the control group. Detailed results comparison are shown in Table 4.

## 4. Discussion

### 4.1. Effectiveness of Training

Pelvic floor muscle training has been proven to be effective in improving LARS symptoms. The results indicated that the degree of completion of training in experiment groups I and II at each time point was higher than that in the control group. Experiment group II combined the stereo-realistic characteristics of 3D animation with the four steps of teach-back (pelvic floor muscle training, method guidance, assessment of mastery, repeated guidance of deficits, confirmation of mastery, and continued exercise), and fully mobilized patients' vision, hearing, and motion perception. Regular health education gives priority to the verbal method of information transmission, usually one way [17, 18]. Teach-back adopts a two-way information transmission mode, to help medical staff to assess patients' understanding of the health education content, through real-time testing of mastery, receiving feedback from the patient, correcting errors, and providing missing information to strengthen and consolidate repeatedly. Its effect was found to be significantly higher than that of conventional health education, and its connotation of health education was quite consistent with the concept of “knowledge-belief-behavior” [19, 20].

LARSS is the most commonly used assessment tool for LARS patients that can evaluate the symptoms of anal exhaust, defecation, tenesmus, and so on. If the score of LARSS is lower, the recovery of anal exhaust and defecation function is better. At 1 month and 3 months after the operation, the total score of LARSS in experiment group II was lower than that in the control group and experiment group I, and the effect at 3 months was better than that at 1 month, indicating that the intervention method in experiment group II obtained stronger results than that in the control group. Realistic 3D animation of the characteristics of pelvic floor muscles provided a visual impact that was memorable and patients could view it in privacy. In combination with the teach-back method, the aim was to emphasis the training content and deepen, preoperative knowledge about pelvic floor muscles, and strengthen this during the postoperative review. It can effectively promote patients to form a deep memory and develop the habit of persistent exercise [21]. Related studies had reported that patients may have temporary uncontrolled defecation in the early stage due to the disorder of normal functioning after anal preservation surgery. However, long-term uninterrupted pelvic floor muscle training of patients was not only conducive to the recovery of normal anal defecation function but also the success rate of treatment of short-term or long-term treatment can reach 50%–92% [22].

The normal rectal and anal sphincter, intact rectal nerve reflex arc, and sound fecal storage mechanism together maintain the normal function of the anus, and the failure of any one of these links leads to various problems [23]. After training, the level of loose stool incontinence and defecation frequency at 1 and 3 months after surgery in group II was better than that of the control group and group I. The reason was that the combination of 3D animation and teach-back on pelvic floor muscle functional exercise made a deep impression in the memory of the patients motivating them to continue exercising for a long time. Abdominal breathing can regulate gastrointestinal peristalsis and intra-abdominal pressure, and the up- and down-training of the muscle can also effectively drive the operation of the levator ani muscle [24]. Kegel exercise can improve the muscle contraction ability of the levator ani muscle and promote the recovery of its innervated nerve function [25]. Fork leg anal levator exercises can further strengthen the intensity of anal levator exercises with the help of hip muscles and thigh strength, inducing effective training of the levator ANI muscle, improving the patient's ability to control stool, and reducing the frequency of defecation [16]. Through perineal four-dimensional pelvic floor ultrasound examination, Yao found that pelvic floor muscle exercise could strengthen the thickness of the levator ani muscle itself and reduce its hiatus, thereby providing strong objective evidence for pelvic floor muscle training to improve the ability of defecation control [26]. In the LARSS items, tenesmus and defecation urgency items in the experiment group II were better than those in the control group and the experiment group I on the 14th day, 1 month, and 3 months after the surgery, and with the increase of time, there were more and more differences between items, indicating that the intervention of the experiment group II was effective. With training through repeated pelvic floor muscle exercises, muscle strength and tension in the region are enhanced along with functioning, promoting the recovery of the sensory nerve endings on the intestinal wall, stimulating and strengthening the excitability of the cerebral cortex which controls defecation, and adding elasticity to the rectum, thereby increasing the storage capacity for excrement and urine and alleviating rectum edema and fibrosis in the surrounding tissue. Thus, the symptoms of tenesmus and urgency of defecation can be improved [27], so as to improve the life comfort of patients.

### 4.2. Limitations and Future Challenges

This study has some limitations. First, limited research time and manpower led to the restriction of the sample to only 94 patients. Furthermore, as it was mainly a hospital intervention, and the follow-up duration was only up to 3 months after the surgery, and there was a lack of continuous nursing guidance for the patients. Moreover, the limitation of research funds, equipment, and other conditions meant that this study did not use an anorectal manometer to measure rectal contraction force and lacked objective evaluation indicators. In the future, we will expand the sample size and the intervention time, study the deficits in patients' home exercise from the perspective of quantitative research, analyze the causes, and add objective indicators involving continuous detection of pelvic floor muscle function.

## 5. Conclusion

This study demonstrated that 3D animation combined with teach-back health education improved the mastery of pelvic floor muscle training and exercise in LARS patients, thereby effectively reducing their symptoms of loose stool incontinence, defecation frequency, tenesmus, and urgency of defecation.

## 6. Implications for Nursing Management

In this study, the combination of 3D animation and teach-back with real-time feedback advantages were applied to the pelvic floor muscle training of LARS patients, which not only promoted the early and rapid recovery of patients but also significantly improved their quality of life, and symptoms of loose stool incontinence, defecation frequency, tenesmus, and urgency of defecation. The results prove that it is worth adopting as a regular course of treatment.

## Figures and Tables

**Table 1:** Comparison of baseline data among the three groups (*n* (%)/$\bar{x}$ ± *s*).

Variable		The control group (*n* = 31)	Experiment group I (*n* = 32)	Experiment group II (*n* = 31)	*χ* ^2^ */F*	*P*
*Gender*	Male	18 (58.10)	20 (62.50)	19 (61.30)	0.138^*∗*^	0.933
	Female	13 (41.90)	12 (37.50)	12 (38.70)		
Age (years)		57.61 ± 7.36	57.03 ± 7.51	56.68 ± 6.89	0.131^#^	0.877
Height (cm)		161.77 ± 5.41	161.25 ± 6.18	162.77 ± 5.10	0.602^#^	0.550
Weight (kg)		55.68 ± 5.93	54.91 ± 5.96	56.81 ± 5.41	0.861^#^	0.426
BMI		21.23 ± 1.56	21.11 ± 1.88	21.43 ± 1.77	0.284^#^	0.753
*Level of education*	Primary school and below	20 (64.50)	17 (53.10)	19 (61.30)	2.824^*∗*^	0.831
	Junior high school	6 (19.40)	7 (21.90)	6 (19.40)		
	High school	1 (3.20)	2 (6.30)	0		
	College degree or above	4 (12.90)	6 (18.80)	6 (19.40)		
*Medical payment method*	New type farmers and residents health care	16 (51.60)	15 (46.90)	13 (41.90)	3.334^*∗*^	0.766
	Residents health	13 (41.90)	14 (43.80)	14 (45.20)		
	Worker health	2 (6.50)	1 (3.10)	3 (9.70)		
	At his own expense	0	2 (6.30)	1 (3.20)		
*Past medical history*	Hypertension	9 (29.00)	10 (31.30)	8 (25.80)	0.634^*∗*^	0.959
	Diabetes	1 (3.20)	2 (6.30)	2 (6.50)		
	No	21 (67.70)	20 (62.50)	21 (67.70)		
*Type of pathology*	Adenocarcinoma	23 (74.20)	25 (78.10)	25 (80.60)	0.378^*∗*^	0.828
	Other	8 (25.80)	7 (21.90)	6 (19.40)		
*TNM staging*	Phase I	6 (19.40)	6 (18.80)	7 (22.60)	5.863^*∗*^	0.439
	Phase II	14 (45.20)	12 (37.50)	7 (22.60)		
	Phase III	9 (29.00)	8 (25.00)	13 (41.90)		
	Phase IV	2 (6.50)	6 (18.80)	4 (12.90)		
*Surgical method*	Laparoscopic assisted surgery	28 (90.30)	27 (84.40)	27 (87.10)	0.501^*∗*^	0.778
	Open operation	3 (9.70)	5 (15.60)	4 (12.90)		

1: ^*∗*^for the *χ*^2^; ^#^for the *F*. 2: TNM staging: *T* (Tumor) represents the size and extent of the primary tumor, *N* (Node) represents regional lymph nodes, reflecting the lymph node metastasis associated with the tumor, and *M* (Metastasis) represents distant metastasis.

**Table 2:** Comparison of training content completion among the three groups (*n*).

Group	Number	Preoperative			On the 7th day after surgery			On the 14th day after surgery			1 month after surgery			3 months after surgery		
		Optimal	Good	Poor	Optimal	Good	Poor	Optimal	Good	Poor	Optimal	good	Poor	Optimal	Good	Poor
The control group	31	3	16	12	4	15	12	6	15	10	5	15	11	4	12	15
Experiment group I	32	12^▲^	14^▲^	6^▲^	13^▲^	13^▲^	6^▲^	15^▲^	13^▲^	4^▲^	14^▲^	14^▲^	4^▲^	11^▲^	13^▲^	8^▲^
Experiment group II	31	14^▲^	11^▲^	6^▲^	15^▲^	11^▲^	5^▲^	18^▲^	11^▲^	2^▲^	15^▲^	13^▲^	3^▲^	12^▲^	13^▲^	6^▲^
*H*	—	9.907			10.405			12.892			11.536			8.824		
*P*	—	0.007^*∗*^			0.006^*∗*^			0.002^*∗*^			0.003^*∗*^			0.012^*∗*^		

1: comparison between groups: ^▲^compared with the control group, all *P* < 0.05. 2: ^*∗*^indicates that there were statistically significant differences between the experiment group I and experiment group II and the control group (*P* < 0.05).

**Table 3:** Comparison of total LARSS scores among the three groups at different time points ($\bar{x}$ ± *s*).

Group	Number	Preoperative	On the 14th day after surgery	1 month after surgery	3 months after surgery	Time effect	Treatment effect	Interaction effect
The control group	31	33.03 ± 3.52	32.42 ± 3.77^Δ^	31.58 ± 4.00^Δ^	30.87 ± 3.77^Δ^			
Experiment group I	32	32.88 ± 4.23	32.22 ± 3.95^Δ^	30.03 ± 3.41	27.50 ± 3.60^▲Δ^	60.732^*∗*^	3.185^*∗*^	10.358^*∗*^
Experiment group II	31	32.71 ± 4.23	31.74 ± 4.07^Δ^	29.65 ± 3.59^▲Δ^	25.48 ± 2.16^◆▲Δ^			
*F*		0.050	0.243	2.167	26.516			
*P*		0.951	0.784	0.047^*∗*^	<0.000^*∗*^			

1: intra-group comparison: ^Δ^ was compared with that before the intervention, all *P* < 0.05. 2: Comparison between groups: ^▲^compared with the control group, ^◆^compared with the experiment group I, *P* < 0.05. 3: ^*∗*^indicates that there were statistically significant differences between the experiment group I and experiment group II and the control group (*P* < 0.05).

**Table 4:** Comparison of LARSS items scores among the three groups at different time points after the operation ($\bar{x}$ ± *s*).

Variable	Group	Number	Preoperative	On the 14th day after surgery	1 month after surgery	3 months after surgery	Time effect	Treatment effect	Interaction effect
Exhaust incontinence	The control group	31	4.87 ± 2.17	4.35 ± 2.17	3.97 ± 1.91	2.77 ± 2.22			
	Experiment group I	32	4.84 ± 2.14	4.47 ± 1.98^Δ^	4.38 ± 1.93^○^	2.66 ± 2.39	42.158^*∗*^	0.138	0.704
	Experiment group II	31	4.77 ± 2.14	4.39 ± 1.96	3.9 ± 1.58	2.32 ± 2.01			
	*F*		0.107	0.026	0.63	0.384			
	*P*		0.983	0.974	0.535	0.707			

Loose stools incontinence	The control group	31	2.81 ± 0.75	2.61 ± 1.02^Δ^	2.52 ± 1.12^○Δ^	2.42 ± 1.20^◇Δ○^			
	Experiment group I	32	2.81 ± 0.74	2.53 ± 1.11^Δ^	2.25 ± 1.32^○^	2.16 ± 1.37^○^	17.682^*∗*^	3.112^*∗*^	4.070^*∗*^
	Experiment group II	31	2.81 ± 0.75	2.42 ± 1.20	1.84 ± 1.49	0.97 ± 1.43^▲◆^			
	*F*		0.001	0.236	2.081	10.404			
	*P*		0.999	0.790	0.131	<0.000^*∗*^			

Defecation frequency	The control group	31	3.61 ± 1.54	3.19 ± 1.35	2.58 ± 1.29	2.45 ± 1.43			
	Experiment group I	32	3.56 ± 1.56	3.00 ± 1.59	2.38 ± 1.56	1.88 ± 1.24	72.448^*∗*^	3.192^*∗*^	3.627^*∗*^
	Experiment group II	31	3.58 ± 1.52	2.65 ± 1.31	1.55 ± 1.23^▲◆^	0.97 ± 1.02^▲◆^			
	*F*		0.009	1.187	4.944	11.268			
	*P*		0.991	0.310	0.009^*∗*^	<0.000^*∗*^			

Tenesmus	The control group	31	14.39 ± 2.38	13.71 ± 3.50^Δ^	13.39 ± 3.51^○^	12.71 ± 4.19^◇○^			
	Experiment group I	32	14.28 ± 2.41	13.47 ± 3.48^Δ^	12.97 ± 4.19^○^	10.94 ± 6.28	18.160^*∗*^	1.549	2.271^*∗*^
	Experiment group II	31	14.23 ± 2.43	13.19 ± 4.25	11.06 ± 6.87	8.65 ± 7.64^▲^			
	*F*		0.036	0.146	1.850	3.349			
	*P*		0.965	0.864	0.165	0.040^*∗*^			

A sense of urgency to defecate	The control group	31	10.55 ± 0.85	10.48 ± 0.89^Δ^	10.13 ± 2.08^○Δ^	9.42 ± 3.25^◇Δ○^			
	Experiment group I	32	10.56 ± 0.84	10.13 ± 1.01^Δ^	9.16 ± 3.14	8.13 ± 4.07^◇^	20.238^*∗*^	5.740^*∗*^	3.507^*∗*^
	Experiment group II	31	10.55 ± 0.85	9.32 ± 2.68^▲^	7.68 ± 4.31^▲^	5.52 ± 5.14^▲^			
	*F*		0.003	3.658	4.326	6.860			
	*P*		0.997	0.034^*∗*^	0.017^*∗*^	0.002^*∗*^			

1: comparison between groups: ^▲^compared with the control group, ^◆^compared with the experiment group I, all *P* < 0.05. 2: intra-group comparison: ^Δ^compared with before intervention, ^○^compared with 14 days after the operation, and ^◇^compared with 1 month after the operation, all *P* > 0.05. 3: ^*∗*^indicates that there were statistically significant differences between the experiment group I and experiment group II and the control group (*P* < 0.05).

## Data Availability

The data that support the findings of this study are available from the corresponding author upon reasonable request.
